# Computation Offloading in a Cognitive Vehicular Networks with Vehicular Cloud Computing and Remote Cloud Computing

**DOI:** 10.3390/s20236820

**Published:** 2020-11-29

**Authors:** Shilin Xu, Caili Guo

**Affiliations:** Beijing Laboratory of Advanced Information Networks, Beijing University of Posts and Telecommunications, Beijing 100876, China; xushilin@bupt.edu.cn

**Keywords:** vehicular cloud computing, remote cloud computing, long short term memory network, deep reinforcement learning, computation offloading, vehicular network

## Abstract

To satisfy the explosive growth of computation-intensive vehicular applications, we investigated the computation offloading problem in a cognitive vehicular networks (CVN). Specifically, in our scheme, the vehicular cloud computing (VCC)- and remote cloud computing (RCC)-enabled computation offloading were jointly considered. So far, extensive research has been conducted on RCC-based computation offloading, while the studies on VCC-based computation offloading are relatively rare. In fact, due to the dynamic and uncertainty of on-board resource, the VCC-based computation offloading is more challenging then the RCC one, especially under the vehicular scenario with expensive inter-vehicle communication or poor communication environment. To solve this problem, we propose to leverage the VCC’s computation resource for computation offloading with a perception-exploitation way, which mainly comprise resource discovery and computation offloading two stages. In resource discovery stage, upon the action-observation history, a Long Short-Term Memory (LSTM) model is proposed to predict the on-board resource utilizing status at next time slot. Thereafter, based on the obtained computation resource distribution, a decentralized multi-agent Deep Reinforcement Learning (DRL) algorithm is proposed to solve the collaborative computation offloading with VCC and RCC. Last but not least, the proposed algorithms’ effectiveness is verified with a host of numerical simulation results from different perspectives.

## 1. Introduction

With the development of artificial intelligence (AI) technology, on-board sensing, and wireless communication technologies, recent years have witnessed a great progress on the intelligent transportation system (ITS). During the process, some resource-intensive safety related intelligent driving technologies are emerging, such as auxiliary driving and autonomous driving [1,2,3]. It is hopeful that people’s hands would be completely liberated in the near future, by which drivers and passengers would have more time and freedom to enjoy a refined and colorful car life. As a result, a host of novel computationally-complex entertainment related vehicular applications have sprung up like mushrooms, e.g., augmented reality (AR), speech recognition and natural language processing, etc. However, the huge computation resource required by the above mentioned vehicular applications poses great challenges to each vehicle’s limited on-board computation resource. The report published by Cisco indicates that 300 million passenger vehicles can generate more than 400 million GB of data in wireless communications [4]. Correspondingly, it can be conceivable that a huge amount of computing tasks could be generated during the process, which can trigger surging requests for massive computation resource. To solve the above challenges, computation offloading has been proposed as a promising approach to alleviate the dilemma of computation resource shortage, which has drawn extensive attentions and research efforts from automakers, software platform providers, and academia. In recent years, considering its important role in future ITS [5,6,7,8,9], a multitude of studies have been devoted to propose effective and efficient computation offloading schemes. Reference [10] concentrates on the collaborations of the different edge computing anchors and puts forward a novel vehicular edge computing framework named as CVEC. Reference [11] puts forward a fog computing- and cloud computing-assisted computation offloading scheme in 5G mobile networks for vehicle-to-grid (V2G) networks. The parked and moving vehicles-based fog vehicular network is proposed to implement computation offloading aimed at the real-time traffic management in Reference [12]. The survey [13] focuses on very recent advances on the current offloading frameworks, computation offloading techniques, and other critical issues.

So far, among the previous computation offloading related works, most of them only focus on remote cloud computing (RCC)-enabled computation offloading schemes, in which the computation tasks are offloaded to the resource-rich infrastructures, such as road side unit (RSU). Although these solutions can theoretically improve service quality, some potential problems have greatly affected their application in vehicular networks in reality. Specifically, the serious delay resulting from long distance transmission, the intermittent wireless communication links arising from dynamically changeable vehicular topology, the limited service coverage due to the inadequate infrastructure deployment, and the expensive costs caused by the investment in infrastructure can directly degrade the performance of RCC enable computation offloading scheme. Moreover, without the utilization of idle on-board computation resource, the RCC-enabled computation offloading scheme can not only cause serious waste of resources, but also can cause serious system failure issues in vehicular scenarios without the deployment of transportation infrastructures. Here, for sake of simplicity, the on-board computation resource-based computation offloading is regarded as vehicle cloud computing (VCC), which is also named as vehicular fog computing (VFC) [14] or mobile edge computing (MEC) [15] in some works. To overcome the aforementioned dilemma, we adopted a comprehensive cross-layer computation offloading framework, which jointly consider the computation offloading on VCC and RCC together. Benefiting from the numerous studies on RCC-based computation offloading schemes, this paper borrows their ideas and will not reinvent the wheel. In this fashion, we focus our attentions on VCC-enabled computation offloading scheme.

In a VCC-based computation offloading scheme, a virtual resource network can be woven with the under-utilized on-board computation resource [16] dispersed on the parked vehicles or the vehicles running on the road, which forms a computation resources pool for computation offloading. To efficiently conduct the collaborative VCC- and RCC-enabled computation offloading, the dynamics and uncertainty on the resource availability of VCC brings huge challenges. In most previous studies, the challenge is usually simplified as a clearly visible computation resource [17], which is unrealistic to real-time exchange on-board resource utilization status in a large-scale vehicular network. In fact, the time-varying and uncertain on-board resource availability can be caused by many factors. Firstly, each vehicle is usually reluctant to share its idle on-board resource with others. The reason for that is caused by the fact that, once agreeing to share resources with other vehicles, one vehicle’s idle resource cannot be flexibly utilized by itself at any time. In addition, in many cases, the resource requirements are not only determined by itself, but some external factors should also be taken into consideration, such as road conditions, traffic accidents, weather conditions, etc. Furthermore, although some vehicles are willing to share their idle computing resource with the motivations driven by some incentive measures, they may cease to share their computation resource with others if only itself surging demand for computation resource. In this way, the VCC-enabled computation offloading is so challenging that an effective resource discovery paradigm is necessary. To solve it, a perception-exploitation computation offloading scheme is proposed, in which resource discovery and computation offloading are jointly investigated.

As for the resource discovery scheme, it is utilized to predict the computation resource utilization status by extracting the temporal correlation of resource utilization status sequence. Specifically, we propose a Long Short-Term Memory (LSTM) network-based resource discovery scheme. With the predictive resource utilization status, the computation offloading is implemented, which formulates a concept of cognitive vehicular network (CVN). In a CVN, vehicles with available resource can be regarded as a primary user, and vehicles with resource requests can be assumed as secondary users. If a secondary user want to utilize the idle resource provided by the primary users, it has to collect historical resource status information from the targeted primary users. With the proposed LSTM model, each vehicle can learn experience from the collected historical data, and the target service vehicles’ resource utilization status can be predicted accurately. On basis of the resource discovery results, an intelligent computation offloading management scheme is necessary to implement the computation offloading under the dynamically changeable vehicular environment. In general, there are centralized and decentralized two types computation offloading management schemes. However, the centralized scheme usually requires massive information exchange among vehicles and expensive computational overhead, which is not practical at all in a large-scale vehicular network. As a result, we adopted a decentralized computation offloading manage scheme. Specifically, in our scheme, the computation offloading management problem is firstly formulated as a multi-constrained knapsack problem, which is NP-hard to be solve with the conventional optimization algorithms. Thereafter, to solve it, the original problem is reshaped as a multi-agent decision making problem, and a novel Deep Reinforcement Learning (DRL) algorithm with a multi-agent iterative updating mechanism is proposed. The main contributions in this work are summarized as follows.

- To conduct the computation offloading for the resource-intensive vehicular applications, the concept of cognitive vehicle network (CVN) is proposed, in which vehicle cloud computing (VCC) and remote cloud computing (RCC) are jointly considered.
- To overcome challenges caused by the dynamics and uncertainty of on-board resource utilization status in a VCC, a perception-exploitation computation offloading scheme is proposed. In the perception stage, a Long Short-Term Memory (LSTM) model-based resource discovery mechanism is designed to predict the on-board computation resource utilization status in a VCC.
- Based on the resource discovery results, a decentralized DRL algorithm-based computational resource allocation scheme is proposed, in which an iterative updating policy is adopted to solve the non-stationary issue and reduce the computation complexity.

The structure of this paper is organized as follows. Section 2 summarizes the related works to our studies. Section 3 describes the system model and formulates the optimization objective problem. Section 4 proposes an LSTM model-based computational resource discovery algorithm. In Section 5, a novel DRL algorithm-based computation offloading management scheme is put forward. In Section 6, numerical simulation results are utilized to verify our algorithms’ effectiveness. Section 7 discusses the novelty and potential problems. The conclusion is summarized in Section 8.

## 2. Related Works

In this section, we report some related works to the relevant concepts and the key technologies mentioned in this paper.

### 2.1. Related Works on Cognitive Vehicular Network

The concept of cognitive vehicular network was proposed some years ago, but, until now, there has been no unified definition. The previous works mainly focus on communication technologies. In Reference [18], a channel access management framework is designed to provide quality of service (QoS) for data transmission in cognitive vehicular networks. In Reference [19], spectrum sensing for opportunistic spectrum access is conducted collaboratively among neighboring vehicles. Reference [20] proposes a new dynamic spectrum allocation algorithm (DSAARCC) to resolve channel conflict problem in channel switching in vehicular network. In Reference [21], an AI approach-based optimal data transmission scheduling scheme in cognitive vehicular networks to minimize transmission costs while also fully utilizing various communications modes and resources. In recent years, the concept of cognitive vehicular network shows a diversified trend. Reference [22] introduces a Cognitive Internet of Vehicles (CIoV) concept to realize intelligent cognition, control and decision-making for future autonomous driving scenarios, in which human-centric CIoV utilizes hierarchical cognitive engines and conduct joint analysis in both physical and network data space. In Reference [23], with a cloud/fog-computing pattern and the internet of things (IoT) AI service framework, a cross-domain solution for auto-driving is proposed in Cognitive Internet of Vehicles. In order to facilitate video streaming application in terms of peak signal-to-noise ratio (PSNR) and smooth playback, Reference [24] proposes a semi-Markov decision process (SMDP)-based resource-allocation scheme. Based on the cognitive engine-based conventional cognitive IoV, Reference [25] formulates the security strategy deployment for switches on core network to meet the safe transmission rules and to obtain the lowest transmission delay. The above work is usually proposed for specific vehicular scenarios or some particular vehicular applications, and the proposed solutions are difficult to extend to other vehicular scenarios and other vehicular applications. Moreover, the concept of cognition in the proposed cognitive vehicular network is diversified, as well. In our proposed cognitive vehicular network, the cognition mainly refers to the perception of resource utilization status, which is crucial to the computation offloading in a VCN. Our research scenario does not limit to a vehicular scenario, and the proposed computation offloading scheme is not aimed at a specific vehicular application. Therefore, its scalability is stronger than other cognitive vehicular networks proposed in the above references.

### 2.2. Related Works on Jointly Computation Offloading Scheme in Vehicular Network

There have been a broad range of studies which are explicitly or implicitly related to our ideas in this paper. Reference [26] proposes a hybrid architecture utilizing the benefits of cloud access networks and edge caching. Reference [27] considers the cooperation of cloud computation and mobile edge computation (MEC) in internet of things (IoT) under the partial computation offloading scheme. Reference [11] puts forward a fog computing- and cloud computing-assisted V2G systems in 5G mobile networks for vehicle-to-grid (V2G) networks. The survey [13] focuses on the recent advances on computation offloading frameworks and the main critical issues existing in the computation offloading techniques. Reference [28] presents an idea of Mobile Vehicular Offloading (MoVeOff), in which data is transferred from on-board devices to mobile devices of drivers and passengers. Reference [29] puts forward a caching strategy consisting of a small-cell cloud and a macro-cell cloud to minimize network latency. Reference [30] comes up with a cooperative vehicular cloud-aided edge caching scheme. Reference [12] formulates a fog IoV system with the parked and moving vehicles for offloading the real-time traffic management. Reference [14] introduces a vehicular fog computing (VFC) with moving and parked vehicles as fog nodes. Reference [10] concentrates on the collaborations among different edge computing anchors and puts forward a novel collaborative vehicular edge computing (CVEC) framework. The above studies mainly involve the collaboration among fog computing, MEC, small-cell cloud and macro-cell cloud. Most of the existing studies assume that all the available computation resource are visible by default, which is unrealistic in some cases. In our model, from a more realistic perspective, only the on-board computation resource utilizing historical information is provided and the real-time resource utilization status is unknown. In our paper, we proposed a CVN concept-based computation offloading scheme. To implement the computation offloading management, a perception-exploitation mechanism is adopted to discover and leverage the computational resource for computation offloading.

### 2.3. Related Works on Resource Allocation for Computation Offloading

The resource allocation for computation offloading generally involve spectrum resource allocation and computation resource allocation. A multitude of studies have devoted to minimizing the total network cost for energy and delay, as well as maximizing the system profit, with the conventional optimization methods, like convex optimization, and game theory, as well as some AI-based machine learning algorithms. Specifically, to maximize the sum offloading rate, a hybrid computation offloading scheme jointly considering the resource allocation integrating task distribution, subchannel assignment and power allocation is proposed in Reference [31], which are solved by subproblems decompose and iterative solution algorithm. To minimize the weighted-sum latency of all mobile devices, Reference [32] formulates a joint communication and computation resource allocation problem to minimize all mobile devices’ weighted-sum latency. Reference [9] proposes a vehicle-assisted offloading scheme for UEs to reduce the computation offloading delay, in which a semi-Markov process is formulated and solve by Q-learning and DRL algorithm. Reference [33] investigates the joint communication and computing resources allocation problems for computation offloading in a vehicular edge computing system. To reduce the long-term expected costs of power and time, Reference [16] discusses the VCC resource allocation problem. In the above works, diverse metrics, such as transmitting rate, delay, energy efficiency, etc., have been considered. In our work, due to the relatively sufficient energy supply in vehicular network, we believe that energy is not the key factor restricting the computation offloading’s performance in a VCN [34]. In addition, considering the requirements for quality of service (QoS) in vehicular network, our optimization objective is defined as maximizing utility function, in view of the powerful data processing ability in complex circumstance and high-effective learning capability from the environment. In this paper, a Deep Q-learning Network algorithm (DQN) is utilized to solve the optimization problems for resource allocation.

### 2.4. Related Works on Computation Resource Utilizing Status Prediction in a VCC Network

In a VCC network, due to the time-varying computation resource availability, it is difficult to know the real-time resource utilization status. Thereby, an effective resource utilizing status prediction scheme can definitely facilitate load balancing and proactive task scheduling in a VCC network. However, to the best of our knowledge, there are few works directly related to resource utilization status prediction, especially the on-board ones. The reasons mainly come from many aspects. Firstly, there is not a completely unified industrial standard for computational resource utilizing in vehicular network. Secondly, the computational resource consumption for some novel vehicular applications, like autonomous driving, security-related and user-oriented vehicular tasks are difficult to accurately and uniformly quantize. Last but not least, the complex road conditions and traffic density, as well as weather variability, are all closely related to computation loads, which is so complicated that till now there are little related studies. In a VCC network, it is very challenging to acquire the knowledge of resource utilization status statistical distribution in advance. Fortunately, some previous research on the prediction of primary users’ behavior in cognitive radio networks (CRN) can give us some important reference and inspiration. Machine learning [35,36,37] has been proven to be a powerful tool for the behavior prediction in CRN, by which a priori knowledge of the distributions is not required. In this work, based on the historical resource utilizing state statistic information, we adopteded a deep learning-enabled LSTM network [38,39,40] to predict the resource availability in a VCC network.

## 3. System Model and Problem Formulation

### 3.1. System Description

**Figure 1:**
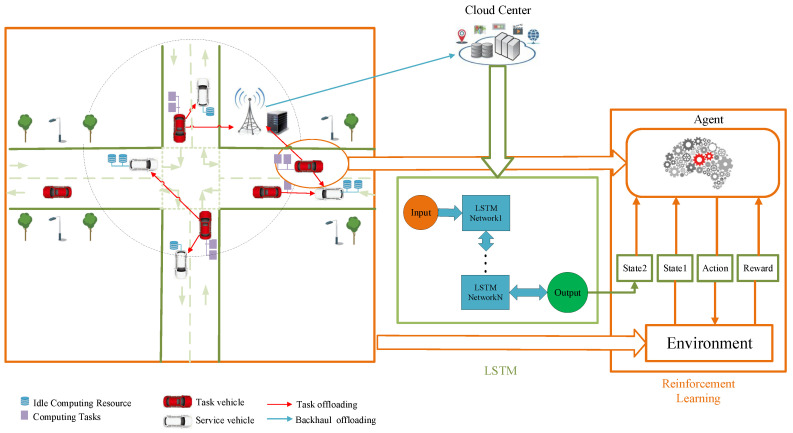
System model for cooperative mobile edge computing and cloud computing.

Our system model is shown in Figure 1. Here, a typical vehicular scenario embraces an unidirectional road with *U* unidirectional lanes is considered. The system mainly embraces three participants: RSU, service vehicles, and task vehicles; their definitions are separately introduced in the following.

- RSU: As a common transportation infrastructure in the vehicular network, it is usually equipped with functions, such as wireless access and vehicular tasks computation. In addition, it is assumed that the RSU is wire-connected with cloud computing center. In this way, we can think that its computing resources are sufficient to meet the needs of offloading computing tasks in the vehicular network. However, because it is usually constructed and maintained by a third-party company, its service price is relative high.
- Service Vehicles: The vehicles with resource availability are defined as service vehicles [41], which can share their limited idle computing resources to other vehicles with resource requests. Since the resource shared by service vehicles are idle, it is reasonable that the service price is lower than RSU.
- Task Vehicles: The vehicles with resource requests are defined as task vehicles [41], which send resource-demanding requests to the neighboring service vehicles or resource-rich RSUs for additional computational resource. They need to pay for the received computation offloading service from RSUs or service vehicles.

It is worth mentioning that the roles of service vehicles and task vehicles are interchangeable in some cases, such as the changing traffic environment and the time-varying vehicular applications. In other words, due to the variability of resource utilization status, service vehicles can be switched to task vehicles, and vice versa. Moreover, the arrivals and departures of service vehicles and task vehicles follow Poisson distribution with parameters $\lambda_{t}$ and $\lambda_{s}$, respectively. Furthermore, the homogeneity of on-board resource distribution is taken into consideration. Here, for notational simplicity, we just assume that each vehicle is equipped with resource requests or availability of one or two resource units (RUs). Since one parked vehicle is stationary, which can be regarded as an RSU node with shrinking resource availability. In addition, there is no parked vehicle in some vehicular scenarios, such as the highway scenario. As a result, in our model we only consider the VCC consisting of the running vehicles on the road.

### 3.2. Communication Model

For computation offloading, both the uploading of computing tasks and the feedback of computing results are closely related to channel quality. As for channel quality, we mainly consider large-scale fading arising form path loss and small-scale fading caused by relative velocity. The large-scale fading $G_{L}$ can be defined as follows.

(1) $$G_{L}=G_{0}{\left(\frac{d}{d_{0}}\right)}^{-\gamma },\;G_{0}=G_{rx}G_{tx}{\left(\frac{\lambda }{4\pi d_{0}}\right)}^{2},$$

where *d* is the distance and γ is the path loss exponent, respectively, $G_{0}$ is the baseline attenuation at the reference distance $d_{0}$, $G_{rx}$, $G_{tx}$ denote the antenna gains, and $\lambda =\frac{c}{f_{c}}$ is the wavelength of carrier frequency $f_{c}$ and light speed *c*. Considering the relative speed in system model, the doppler effect-based small scale fading is not negligible. In (2), it is demonstrated by rayleigh channel model with parameters *z* and σ.

(2) $$f\left(z\right)=\frac{z}{\sigma^{2}}\exp\left(-\frac{z^{2}}{2\sigma^{2}}\right).$$

In order to perform computation offloading, the Device-to-Device (D2D)-based vehicle-to-vehicle (V2V) links, Non-orthogonal multiple access (NOMA)-based vehicle-to-multiple-vehicles (V2mV) links, and vehicle-to-infrastructure (V2I) links are considered in our model. In addition, each link utilize the spectrum resource in orthogonal frequency division multiple access (OFDMA) mode. In this way, there is no interference caused by spectrum resource sharing. Above all, the aforementioned communication links are detailed as follows.

- V2V links: Their capacity can be defined with (3). Without the participation of central control unit and reliance on the assistance of transport infrastructure, V2V links is utilized for free.
  (3) $$C_{i,j}=B\left(\log_{2}\left(1+\frac{P_{i,j}{\left|h_{i,j}\right|}^{2}}{N_{0}}\right)\right).$$
- V2I links: Here, one task vehicle offloads its computing task to clouding computing empowered transport infrastructure, like RSUs., and its capacity is the same as V2V links in (3). V2I links need to pay for the assistance of transport infrastructure.
- V2mV links: To satisfy the requirements of multi-terminal access, the concept of V2mV link has been proposed in our previous work. Here, considering the delay and complexity caused by successive interference cancellation (SIC) technology-enabled decoding technology, it is assumed that each task vehicle can offload its computational tasks to at most two destinations. The power allocation scheme is shown in (4). Under the constraint of transmitting power $P_{i}$, when channel gain $h_{i,k}$ between vehicle *i* and *k* is inferior to $h_{i,k^{\prime }}$ between vehicle *i* and $k^{\prime }$ in (4), power $P_{i,k^{\prime }}$ allocated to vehicle $k^{\prime }$ is more than power $P_{i,k}$ allocated to *k*. Thereafter, the capacity of V2mV is presented in (5)–(7).
  (4) $$h_{i,k}<h_{i,k^{\prime }}\to P_{i,k}<P_{i,k^{\prime }},where\;P_{i,k}+P_{i,k^{\prime }}\leq P_{i},$$
  (5) $$C_{i,k}=B\left(\log_{2}\left(1+\frac{P_{i,k}{\left|h_{i,k}\right|}^{2}}{I_{i,k}^{Intra}+N_{0}}\right)\right),$$
  (6) $$C_{i,k^{\prime }}=B\left(\log_{2}\left(1+\frac{P_{i,k^{\prime }}{\left|h_{i,k^{\prime }}\right|}^{2}}{N_{0}}\right)\right),$$
  (7) $$C_{i}=C_{i,k}+C_{i,k^{\prime }}.$$

### 3.3. Computation Model

In our model, we mainly consider the resource availability on service vehicles and RSUs. It is assumed that the available resource provided by RSUs are stable and known in advance. However, service vehicles’ resource utilization status are full of uncertainty for the following reasons.

- Due to selfishness and privacy, service vehicles are reluctant to share their idle computing resource with other vehicles. As a result, the task vehicles cannot efficiently obtain the service vehicles’ real-time resource utilization status.
- After receiving the resource requests from their neighboring task vehicles, the service vehicles can decide to refuse or accept these requests with their willingness.
- Even if these requests are accepted, the ongoing offloading service have to be ceased once the service vehicle itself resource demanding is abruptly surging.

Based on the above analysis, resource discovery is crucial to the computation offloading in a VCC network, in which the computation resource is dynamic and uncertain. With the help of resource discovery, Network Function Virtualization (NFV) technology is utilized to collect the dispersed computational resource and generate a virtual cloud computing resource pool. Moreover, with software-defined networking (SDN) technology, communication and computation resource can be efficiently managed to enhance the performance of CVN [42]. As for the computation offloading management, we firstly assume that computation tasks can be divided into several subtasks with unit computational complexity, and the partial offloading and parallel computing can be supported by a two-tier offloading framework [43]. The computational resource scheduling and control can be conducted by a centralized or distributed approach. Due to the self-organized characteristic in computation offloading, we adopted the distributed computation offloading scheme in this paper.

### 3.4. Problem Formulation

In this section, the computation offloading in a CVN can be formulated as a multi-variable multi-constraint combinatorial optimization problem as (8).

(8) $$\max R^{total}\left(L,l,P\right),$$

$$s.t.\left\{\begin{array}{c} C1:\sum_{1\leq j\leq K^{s}}\sum_{1\leq i\leq K^{t}}L_{i}^{j}C_{i}^{j}\leq \sum_{0\leq j\leq K_{s}}C_{j}^{comp},\\ C2:\sum_{0\leq j\leq K^{s}}L_{i}^{j}C_{i}^{j}\leq C_{i}^{comp},\\ C3:\sum_{1\leq i\leq K^{t}}L_{i}^{j}C_{i}^{j}\leq C_{j}^{comp},\\ C4:\sum_{0\leq i\leq K^{t}}L_{i}^{j}\leq N_{1},\\ C5:\sum_{0\leq j\leq K^{s}}L_{i}^{j}\leq N_{2},\\ C6:\sum_{0\leq j\leq K^{s}}l_{i}^{j}\leq 1,\\ C7:\sum_{0\leq j\leq K^{t}}L_{i}^{j}P_{i}^{j}\leq P_{i}, \end{array}\right.$$

wherein *L* denotes the computation offloading decision, *l* is the spectrum allocation results, and *P* is the power allocation results. $C_{i}^{j}$ is the offloading tasks from task vehicle $i,1\leq i\leq K_{t}$ to service vehicle $j,1\leq j\leq K_{s}$ or RSU j=0. As for the constraints, C1 denotes that the overall allocated computation tasks should not exceed the overall resource availability $\sum_{0\leq j\leq K_{s}}C_{j}^{comp}$. C2 states that the allocated resource for each task vehicle $i,1\leq i\leq K_{t}$ should not surpass it requesting resource. C3 means that the allocated resource provided by each service vehicle $j,1\leq j\leq K_{s}$ should not surpass its resource availability. C4 defines $N_{1}$ as the maximal amount of task vehicles served by each service vehicle *j*. C5 defines $N_{2}$ as the maximal service vehicles amount allocated to each task vehicle. C6 means that each link should be allocated to at most one spectrum resource block. C7 is the task vehicle’s power allocation constraints. As aforementioned, the power is relatively sufficient in a VCN, and each link access the spectrum resource with the OFDMA mode. In this way, the optimization can reduce from $\left\{L,l,P\right\}$ to *L*, and the jointly computing resource and communication resource allocation will be investigated in the future work.. To solve the objective function (8), the resource discovery and computation offloading management are, respectively, investigated in our model. In (8), maximizing the overall utility value $R^{total}$ is defined as the optimization objective, which mainly embraces computation utility $R^{comp}$ and communication utility $R^{comm}$ with the weight $\lambda_{1}$ and $\lambda_{2}$, respectively.

(9) $$R^{total}=\lambda_{1}R^{comm}+\lambda_{2}R^{comp},\;R^{comm}=\sum_{1\leq i\leq K^{t}}R_{i}^{comm},\;R^{comp}=\sum_{1\leq i\leq K^{t}}R_{i}^{comp}.$$

## 4. LSTM-Based Resource Discovery in a VCN

As mentioned before, resource discovery is an effective approach to alleviate the dynamics and uncertainty of resource availability in a CVN. In this section, we utilize the collected historical information of resource utilization status to predict the available resource in the future. Till now, the related works on resource utilization status prediction is few so far. Due to the aforementioned similarity between CVN and the conventional cognitive radio network (CRN), the spectrum utilization status prediction in a CRN can provide important reference to our research. Specifically, in a CRN, there mainly exist two participants: primary users (licensed users) and secondary users (unlicensed users). Primary users can occupy the licensed spectrum with a higher priority than secondary users. At each time slot, the spectrum utilizing status (busy or idle) on a specific channel is determined by the appearance of primary users or not. In the channel utilization status prediction mechanism, secondary users firstly predict the primary users’ behaviors and, based on the channel status, to opportunistically access the idle spectrum.

In our model, similar to the idea that channel status prediction mechanism, service vehicles can be regarded as primary users and the task vehicles correspond to secondary users in CRN. In addition, we assume that each vehicle’s historical on-board resources utilization status information can be collected by other vehicles with the transmission on the idle channels. Based on the collected historical information, the coming resource utilizing status at next time slots can be predicted, which paves the way for the subsequent computation offloading management. In the following, we will firstly overview some preliminary knowledge in a CRN, and progressively transfer from CRN to CVN by the comparisons between CVN and CRN. Generally speaking, although the concepts of CRN and CVN are similar in many respects, there are still many differences between them. Due to the environmental complexity in a CVN, the resource utilization status prediction in a CVN is more challenging than the channel status prediction in a CRN. The main differences are summarized as follows:

- Firstly, different from channel utilization status prediction in a CRN, so far there is no effective approached to track the dynamic variability of on-board computation resources. In addition, there is not a central control unit or an unified coordination mechanism among vehicles.
- Secondly, the resource sharing policy in a CVN is fundamentally different from spectrum sharing principle in a CRN. Unlike the spectrum sharing with a predefined tolerable interference threshold in a CRN, the available resource is limited and it can only be shared with restricted amount of vehicular tasks.
- Last but not least, the CRN environments for channel status prediction are usually relatively static, whereas the CVN environment for resource utilization status prediction is highly dynamic. As a result, the resource discovery in a CVN is more challenging than the channel status prediction in a CRN.

Based on the above reasons, the conventional algorithms for primary users’ behaviors prediction in a CRN cannot be straightforwardly utilized to perform resource utilization status prediction in a CVN, such as Linear Prediction, Hidden Markov Decision Model (HMM), Support Vector Machine (SVM), etc. [44]. With the progress of AI technologies, we adopted the deep learning-based Long Short-Term Memory (LSTM) network to implement the resource discovery in a CVN. Owing to the difficulty in collecting the real data set, the self-similarity traffic simulator is adapted to simulate the resource utilization status in a CVN. In the following, the self-similarity traffic model-based computational resource status simulator is firstly presented. After that, the specification of LSTM-enabled resource availability prediction scheme is presented.

### 4.1. Self-Similar Traffic Simulator

As mentioned before, due to the constraints on hardware level and heterogeneous on-board resource configuration, it is challenging and unrealistic to obtain the real data set for resource utilization status prediction in a VCN. Considering the similarity between network traffic and the arrival of vehicular tasks, we adopted a self-similar process-based network traffic model to generate the required simulation data set. Specifically, as for network traffic model, it is one of key issues for network performance management, QoS management, and admission control. So far, there mainly exists three types of traffic models, namely Poisson traffic, Interrupted Poisson (IP) traffic, and Self-similar (SS) traffic, which can characterize the "self-similarity" statistics property of the network traffic. Since the coming computation tasks in a CVN usually obey a potentially complex distribution, which is similar to the traffic of network requests. Among the several network traffic models mentioned above, Poisson traffic can only generate a relative simple time series with short similarity, and which cannot characterize the complex vehicular tasks. Due to the limitations of model framework, IP can simulate more complex vehicular scenario than Poisson traffic model. However, it is incapable of presenting the burst and self-similarity property in real network traffic. SS traffic model is more powerful than both Poisson traffic model and IP traffic model, which has been widely utilized to present the stochastic processes with self-similarity characteristics. Specifically, SS is a well-known characteristic in the domain of internet traffic, and whose main traits is long range dependence of traffic, burstiness and correlation over varying time scales. In self-similar network model, SS is employed to generate the self-similar traffic with Pareto distribution, and its PDF is defined as (10). As a result, we adopted SS traffic to simulate the computation resource utilization status in our model.

(10) $$f\left(x\right)=\beta \frac{\alpha^{\beta }}{x^{\beta +1}},\;x\geq \alpha ,$$

where β>0 denotes the shape, and α>0 signifies the distribution’s scale which is the minimum value of variable *x*. Its mean is given in (10) when the shape parameter β>1. In addition, its variance for variable *x* is infinite when β≤2. The hurst is short for *H* and given in (11), which can quantify SS traffic and reflect self-similarity when $H\;\in \;\left(0.5,\;1\right)$. On condition that H=0.5, the Pareto distribution is simplified to a Poisson distribution, and the burstiness in the traffic with the increase of *H*. Pareto distribution is the simplest heavy tailed distribution, and $\beta_{on}$ and $\beta_{off}$ are parameters about the heavy tailed property of “O” state and “OFF” state, respectively. (11) is the ideal result for burst *H*, whereas the usual value of β is between $\beta_{on}$ and $\beta_{off}$ in reality.

(11) $$H=\frac{3-\beta }{2},\;\beta =\min\left(\beta_{on},\beta_{off}\right),$$

**Figure 2:**
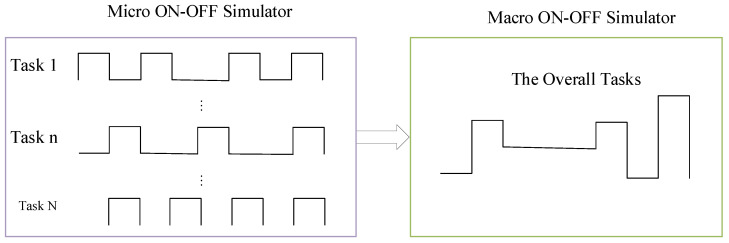
The ON/OFF Simulator Model.

Next, referring to the above model, we utilize ON/OFF model to generate the expected data set with Pareto distribution. To achieve that, we will firstly make the following assumptions: (a) for each type vehicular computing task, its arrival and departure is usually assumed to obey Poisson distribution, and (b) multiple vehicular tasks are independent processes with their own distributions, respectively. Then, Taqqu et al.’s research results have theoretically proved that the superposition of an infinite number of independent update-return processes with heavy-tailed distributions converges weakly to the typical self-similar process fractal Brownian motion (FBM) [45]. Here, the update and return process refers to the ON/OFF process in the Packet-Train model, which has strictly alternating ON and OFF states with their, respectively, own heavy-tailed characteristics. In view of the above concepts, we can explain it from both macro and micro perspectives. From a macro-perspective, the status ON represents the available on-board resource can support the coming computation tasks, while the status OFF indicates the opposite of the status ON. From a micro-perspective, the status ON represents that vehicular tasks are continuously generated at a constant rate, while the status ON indicates that there is no new generating vehicular tasks. In Figure 2, the relationship between the macro model and the micro model is clearly demonstrated. In fact, the macro version can be regarded as an accumulated version of the micro version.

To superimpose multiple independent ON/OFF service sources to generate computation tasks with self-similar characteristics. The heavy-tailed distribution of the ON/OFF service source is represented by a Pareto distribution given in (12).

(12) $$x=\frac{\alpha }{u^{1/\beta }},$$

where *u* is an real number which obeys the Uniform distribution between 0 and 1, and α is short for $\alpha_{on}$ or $\alpha_{off}$ which represent the minimal value of $T_{on}$ or $T_{off}$, respectively. β is the heavy-tailed property parameter. Here, the period for both ON state and OFF state can be achieved by continuously generating random number and feed into (13). On condition that β>1, the mathematical expectation of *x* is defined in the following formula:

(13) $$\bar{T}=E\left(x\right)\;=\frac{\beta \alpha }{\beta -1}.$$

The minimum value $\alpha_{on}$ of ON state is the consuming time for completing a computing task, while the minimum value $\alpha_{off}$ of OFF state can be determined by the time span ratio $T_{ratio}$ in (14), and $\alpha_{off}$ is assumed to be identical for different types of computing tasks. Based on the above analysis, the whole process about generating the vehicular computing tasks sequence is given in Algorithm 1.

(14) $$T_{ratio}=\frac{mean\left(T_{on}\right)}{mean\left(T_{on}\right)+mean\left(T_{off}\right)}=\frac{\frac{\beta_{on}\alpha_{on}}{\beta_{on}-1}}{\frac{\beta_{on}\alpha_{on}}{\beta_{on}-1}+\frac{\beta_{off}\alpha_{off}}{\beta_{off}-1}}=\frac{\frac{\alpha_{on}}{T_{on}}}{\frac{\alpha_{on}}{T_{on}}+\frac{\alpha_{off}}{T_{off}}}.$$

**Algorithm 1** The Computation Resource Tasks Sequence with Self-similar Traffic Model**Input:** Define the computing task types $\left[1,\;\cdots ,\;N\right]$, system parameters β, $T_{on}$, $T_{off}$, $\alpha_{on}$, $\alpha_{off}$
**Output:** The cumulative computing tasks sequence

 1: **for all**
$\;n\;\in \;\left(1,\;\cdots ,\;N\right)\;$
**do**

 2:  Initialization $\beta^{n}$, $T_{on}^{n}$, $T_{off}^{n}$, $\alpha_{on}^{n}$, $\alpha_{off}^{n}$

 3:  **for all**
$\;time\;\in \;\left(1,\;\cdots ,\;Times\right)\;$
**do**

 4:   Generate random number *u* with Uniform distribution between 0 and 1

 5:   **if**
 flag = 0 
**then**

 6:    Achieve the period $T_{on}^{n}$ with (13) 7:    Generate the computation tasks continuously within $T_{on}$ 8:    Set flag=1 9:   **else** 10:    Achieve the period $T_{off}^{n}$ with (13) 11:    Keep sleep within $T_{off}^{n}$ 12:    Set flag=0 13:   **end if**
 14:  **end for**
 15: **end for**

 16: **return** The cumulative computing tasks sequence

### 4.2. LSTM-Based Resource Utilization Status Prediction in a VCN

With the aforementioned SS Traffic simulator, the generated simulation data set can be divided into the training data set and testing data set for the resource discovery algorithm proposed in this section. The prediction accuracy poses a significant influence on the performance of computation offloading in a CVN. Thereafter, an efficient prediction paradigm should be put forward to conduct the resource discovery for each vehicle. The resource discovery issue in a CVN is a typical Time Series Forecasting (TSF) problem [46]. There have been a lot of conventional schemes implementing the time series analysis prediction, such as support vector machine (SVM) [47,48], probabilistic graphical models, HMM [47,49,50], Bayesian Network [51] and random forest classifiers [52], etc. Due to the powerful data processing ability, machine learning has been widespread used in recent years. In particular, with the significant advances of artificial neural networks (ANNs), some deep learning-based models, like recurrent neural network (RNN) [53,54], have been proposed for prediction and gradually become a trend. However, for the prediction of long-range time series, the backpropagation process of RNN suffers from the vanishing [55] or exploding gradient issues. As a result, we adopted LSTM network to conduct the prediction of resource utilization status in a CVN.

**Figure 3:**
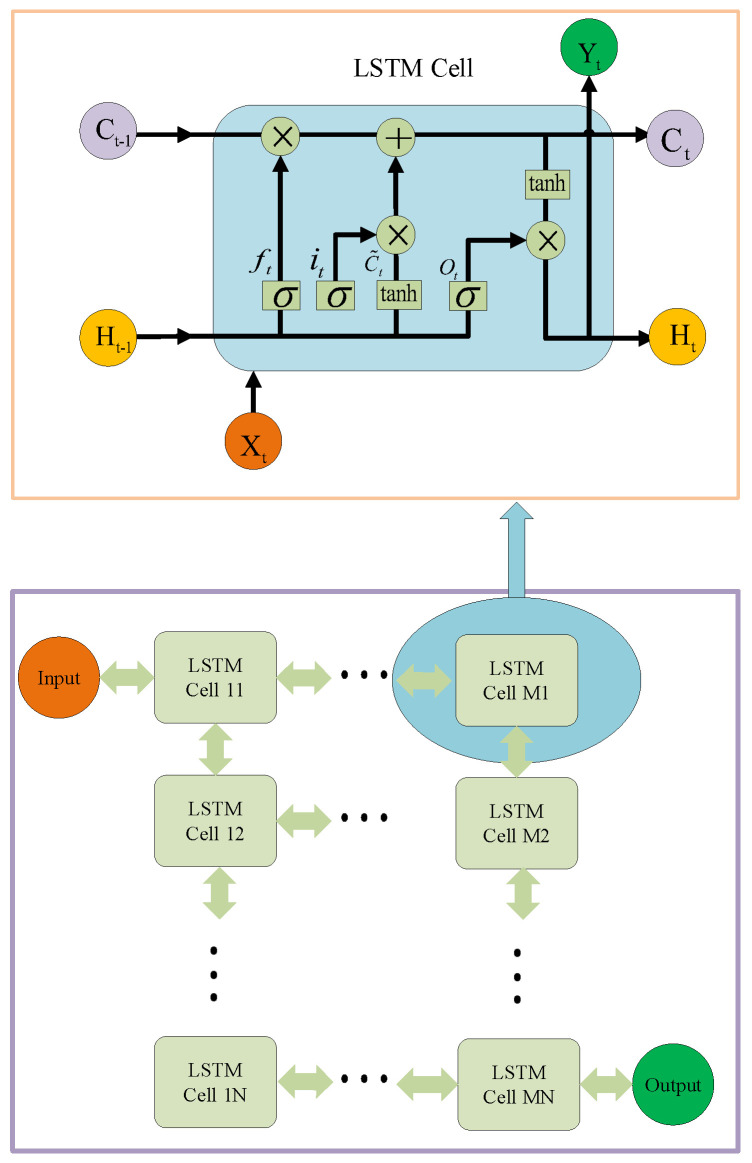
The framework of Long Short-Term Memory (LSTM) network.

Compared with the conventional RNN framework, LSTM is more effective to extract the temporal correlation existing in the temporal sequence, which mainly benefits from the loop framework to remember the time-correlated sequence. Specifically, the standard LSTM module compromises four gate framework-based LSTM cell, by which LSTM is emdowed with the long-term memory. Specifically, the gate framework are a arrange of memory units spanning from previous slots $S_{t-1}$ to the current slot $S_{t}$ cascaded through a cell state, which has been shown in Figure 3. Specifically, the number of units is related to length of time sequences to be studied. For each LSTM cell, which usually embraces three gate activation functions $\sigma_{1},\sigma_{2}$ and $\sigma_{3}$, paired with two output activation functions $\phi_{1}$ and $\phi_{2}$. The framework for neural network is related to the complexity of the specific problem, which can be experienced determined with repeat tests.

(15) $$f_{t}=\sigma \left(W_{f}.\left[H_{t-1},X_{t}\right]\right)\;+\;b_{f}),$$

(16) $$i_{t}=\sigma \left(W_{i}.\left[H_{t-1},X_{t}\right]\right)\;+\;b_{i}),$$

(17) $${\tilde{C}}_{t}=\tanh\left(W_{C}.\left[H_{t-1},X_{t}\right]\;+\;b_{C}\right),$$

(18) $$C_{t}=f_{t}*C_{t-1}\;+\;i_{t}*{\tilde{C}}_{t},$$

(19) $$O_{t}=\sigma \left(W_{o}.\left[H_{t-1},X_{t}\right]\;+\;b_{o}\right),$$

(20) $$H_{t}=O_{t}*tanh\left(C_{t}\right).$$

It is obvious that (15)–(17) and (19) usually have the same input vector $H_{t-1},X_{t}$, which are the hidden state and the temporal sequence input. (15) denotes the forget gate, which can directly decide the remaining portion of the previous cell state $C_{t}$, wherein $W_{f}$ is the weight of neural network. (16) is the input gate which is related to the portion of the current cell state ${\tilde{C}}_{t}$, wherein $W_{i}$ is the weight of neural network. (20) can generate the current state which is independent to the previous cell state. Based on (15)–(17), the combination of the previous cell state and the current cell state can be obtained with (18). (19) is the output gate which can output the result of the cell unit. (20) can generate the hidden state $h_{t}$ in the current time. It is noteworthy that the current cell unit state ${\tilde{C}}_{t}$ and the present hidden state $h_{t}$ will transmit to the next cell unit as its previous states. Like standard back propagation, the LSTM network is trained with backpropagation through time (BPTT), which comprises a repeated application of the chain rule. In our paper, the proposed LSTM model is designed as a two-layer deep learning framework with 64 units on each layer. Correspondingly, the input time sequences are vectors with the dimension 1×64. The output result is the prediction value for the next time slot, and there is no need for activation in output layer. In the training stage, time back propagation transmitting is utilized to iteratively update the weight value of multi-layer neural networks.

## 5. Multi-Agent Double Deep Q Network (DDQN) Algorithm for Computation Offloading in a CVN

### 5.1. The Multi-Agent DRL Framework

In this section, based on the resource discovery results in a VCN, an efficient offloading policies is necessary and the conventional algorithms cannot be competent for the tricky problems or the computational complexity is unacceptable. Fortunately, machine learning has been envisioned as a promising paradigm for addressing such challenges and competent for resource scheduling in complex environments [56]. By virtue of intelligent environmental interaction and self-decision ability, it can facilitate in-depth feature discovery and conduct a flexible and adaptive resource management. In this paper, the DRL-based Double Deep Q Network (DDQN) is adopted. Generally speaking, to implement the computation offloading in a VCN, the centralized and the distributed paradigms have been investigated. In most cases, the centralized scheduling scheme highly relies on the global information, which can cause a heavy communication burden. As a result, we adopted the distributed resource allocation scheme in this paper, which is practically feasible and computationally efficient.

To perform the computation offloading in a CVN, each task vehicle is defined as an agent. Due to the multiple vehicles’ participation, a multi-agent environment is formulated, in which each agent makes a decision based on its local environmental observation. The simultaneous behaviors conducted by all the agents can cause a non-stationary computation offloading environment. As a result, it is hard or even impossible to reach a Nash equilibrium for all the agents. To solve it, we innovatively decompose the multi-agent simultaneously update to sequentially update. In other words, the environment will change from all agents update together to iteratively change one by one. Different from the conventional DDQN algorithm, the iteration sequence of the agent is a problem worthy of in-depth studies in the future. In this paper, to verify the effectiveness of our proposed idea, diverse participation sequence schemes have been taken into consideration.

In addition, another highlight in our paper is the concept of local state space and action space, which can reduce the action space by removing those action sets with a very low probability, thereby reducing the computational complexity significantly. Here, we take the action space as an example to illustrate the reason for why we choose local space instead of global space. In particular, the action space represents the candidate task vehicles set to be chosen for computation offloading. Account for the virtualized computing resources, it can break through the limitations brought by hardware architecture and implement the computing tasks separately. In this way, the role of computational resource in different service vehicles are almost the same for one task vehicle. Then, the computation offloading performance is mainly depends on the communication stage. If the service vehicle is far from the task vehicle, the transmission rate will be inevitably deteriorated due to the long distance path loss. As a result, in order to maximize its own profit, each vehicle will have extremely low probability of choosing a service vehicle in a distance and the service vehicle in proximity are contained in the state space and action space is sufficient for achieving a acceptable offloading scheme. Additionally, it is worth mentioning that the amount of concerned neighboring vehicles is the same for each agent for notational simplicity.

Due to the Markov property, the resource allocation strategy can only be determined by the current state. As a result, the original problem can be simplified as a Partially Observed Markov Decision Process (POMDP) problem, which can be defined as a tuple $\left(S,A,R,T,\gamma \right)$ with the presentation of state space, discrete action space, state transition probability and discount factor along with reward. In the following, we will define these components in our framework, respectively.

State Space: The state space $S_{i}$ for task vehicle *i* is given in (24), which can be regarded as a features vector extracted from the vehicular environment. In $S_{i}$, $B_{i}$ and $V_{i}$ are vehicle *i*’s position and velocity, $\Lambda_{i}$ represents its neighbor vehicles set for task vehicle *i*. $T_{i}$ and $F_{i}$ are the required computational resource and the demanding spectrum resource, respectively.

(21) $$S_{i}\;=\;\left\{B_{i},V_{i},\Lambda_{i},T_{i},F_{i}\right\},\;S_{i}\in S$$

Action Space: The action space is composed of the candidate computation offloading targets, such as service vehicles and RSUs. Moreover, in order to reduce the dimensionality of the action space, a shrinking action space consisting only of the neighbor service vehicles. The intuition behind our idea is that, due to the advantage of short distance in transmission performance, each task vehicle usually chooses the service vehicles in the proximity as its offloading destinations. Moreover, with the proposed neighbor agents mechanism with a uniform size, the scalability can be enhanced to adopt the vehicular network with a dynamically changeable vehicles.   

(22) $$A_{i}\;=\;\left\{L_{i}\right\},\;L_{i}\;=\;\left\{L_{i}^{0},L_{i}^{1},\;\cdots ,\;L_{i}^{K^{t}}\right\}.$$

Reward Function: Reward function is the driving force of approaching the optimization objective in (9). Here, $R^{total}$ is defined as the overall reward in our model, which mainly embraces the reward arising from communication stage $R^{comm}$ and computation stage $R^{comp}$. Specifically, $R^{comm}$ can be jointly determined by the benefits and costs of communication stage as (23). In (23), $benefit^{comm}$ is the total transmission rate consisting of three types of links: V2V, V2I, and V2mV. $cost^{comm}$ is the cost paid by the V2I links, while α is the unit price for the transmitting rate of V2I links.

(23) $$R^{comm}=benefit^{comm}-cost^{comm},\;benefit^{comm}=C^{V2V}+C^{V2I}+C^{V2mV},\;cost^{comm}=\alpha C^{V2I}.$$

As for computation stage, it is assumed that service vehicles voluntarily shares their underutilized resource to task vehicles without charging the payments. As a comparison, the computation resource equipped on RCC needs the task vehicles’ additional payment. Here, $R^{comp}$ is defined with (24), which is jointly determined by the benefit $benefit^{comp}$ and cost $cost^{comp}$, as well.

(24) $$R^{comp}=benefit^{comp}-cost^{comp},\;benefit^{comp}=\sum_{1\leq i\leq K^{t}}\sum_{0\leq j\leq K^{s}}L_{i}^{j}C_{i}^{j},\;cost^{comp}=\beta \sum_{1\leq i\leq K^{t}}L_{i}^{0}C_{i}^{0},$$

wherein β is the unit price for computation resource provided in RC, $L_{i}^{j}=1$ indicates the task vehicle *i* offload its computation tasks to service vehicle $j,1\leq j\leq K^{s}$ or infrastructure j=0, and, otherwise, $L_{i}^{j}=0$, $C_{i}^{j}$ is the task vehicle *i* obtained computation resource from service vehicle $j,1\leq j\leq K^{s}$ or infrastructure j=0.

As for the previous studies on computation offloading management issues in vehicular networks, only some objective factors are taken into consideration, like vehicles’ positions, speeds, channel state information (CSI), and computation resource requirements. Here, in addition to the objective factors, some subjective factors are taken into account, as well. In our model, the subjective factors mainly refer to the selfish level of service vehicles for resource sharing with others. $\phi =\left\{\phi_{0},\phi_{1},\cdots ,\phi_{j},\cdots ,\phi_{K^{s}}\right\}$. $\phi_{0}=1$ represents that RSU has the same attitude towards all task vehicles. For service vehicle $j,1\leq j\leq K^{s}$, its attitude towards task vehicles are defined as $\phi_{j}=\left\{\phi_{j,1},\cdots ,\phi_{j,i},\cdots ,\phi_{j,K^{s}}\right\}$, wherein $\phi_{j,i}\in \left[0,1\right]$. Above all, $R^{total}$ can be obtained as follows.

(25) $$R^{total}=\sum_{0\leq j\leq K^{s}}\sum_{1\leq i\leq K^{t}}\phi_{j,i}\left(\lambda_{1}R_{i,j}^{comm}+\lambda_{2}R_{i,j}^{comp}\right).$$

Asynchronous Iteration Sequence: As mentioned before, the iteration sequence is one of characteristics of our proposed algorithm, which can be determined by a swarm of predefined principles. In fact, the principles can be adaptively adjusted based on environmental perception, or be customized according to the vehicular scenario’s requirements. In order to reflect the effectiveness of asynchronous iteration sequence, in our model, we only propose a few representative schemes, such as location-based iteration order- and value-based iteration order, etc.

### 5.2. Multi-Agent DDQN-Based Computation Offloading Management Scheme

In a CVN with a large amount of vehicles, the extraordinary computation complexity brings huge challenges to the delay-sensitive vehicular network. As a result, the optimization algorithm should be elaborately designated to manage computation offloading. With the progress of reinforcement learning (RL), several versions of RL algorithms have been proposed, such as Q-learning, Deterministic Policy Gradient (DPG), and their variants. Generally speaking, from the perspective of designed principles, the above RL algorithms can be divided into two types: value iteration or policy iteration. Q-learning is a tabular-based value iteration algorithm, in which a Q-table should be maintained and updated during the learning process. Due to the expensive memory required by the Q-table, Q-learning can only be utilize to solve the small scale problems. DPG is a policy iteration-based RL algorithm, which is endowed with a powerful learning ability to solve the complex problem with a high-dimensional action space. However, due to the extraordinarily huge solution space, DPG algorithm usually has extraordinary computation complexity. In this way, a deep Q-learning network (DQN)-based RL algorithm has been proposed in recent years, which combines the benefits of Q-learning and Deep learning.

**Figure 4:**
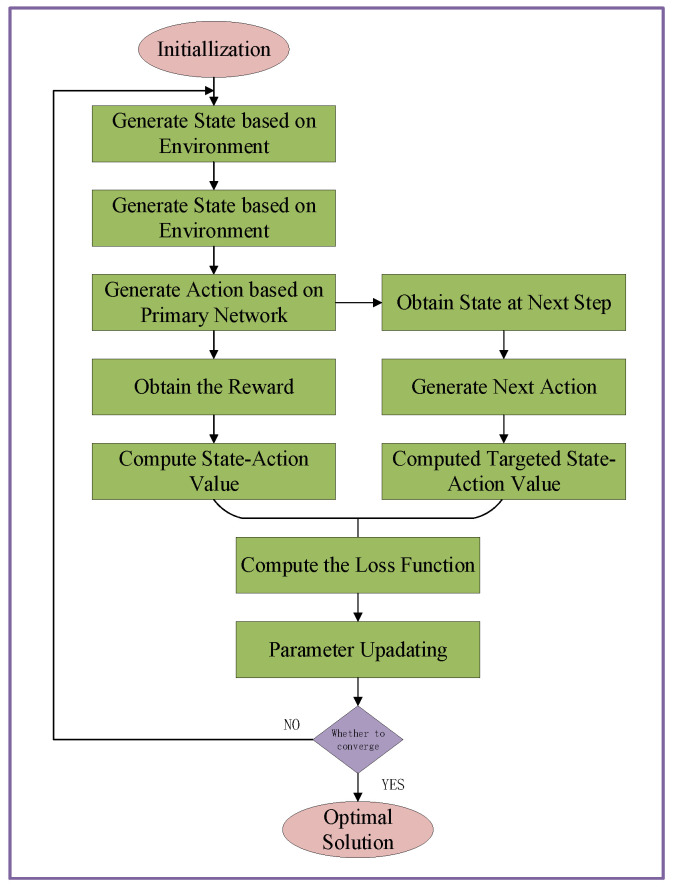
The flow chart of Deep Reinforcement Learning (DRL)-based Double Deep Q Network (DDQN) Algorithm.

As for the DQN algorithm, a value approximation function enabled by deep learning to replace the Q value stored in the Q-table. Nevertheless, a fact that cannot be ignored is that DQN still cannot overcome the overestimation issue of target value existing in Q-learning algorithm. To solve it, a DQN-based double deep Q-learning algorithm is adopted to implement the computation offloading management in a VCN. As an advanced version of DQN algorithm, DDQN algorithm utilize experience replay and separate target network framework to solve the overestimation issues. Specifically, with the separate target network framework, the target network’s parameters is unchangeable within a per-defined times span. As a result, the chosen action by the target network can endow a more conservative target value, and the overestimation issue can be alleviated to a certain degree. In the training stage, the training instability can seriously affect system performance, especially in the multi-agent environment. For each agent, its training environment is unstable due to the simultaneous updating process of multiple agents. Fortunately, the dilemma can be effectively solved with the proposed asynchronous iteration sequence mechanism. Next, the flow chart of the proposed multi-agent DDQN algorithm is summarized in Figure 4, and more detail is given as follows.

Initialization:At the beginning, the primary network $Q\left(s,a;\theta \right)$’s parameters $\theta^{0}$ and the target network $Q\left(s,a;\theta_{-}\right)$’s parameters $\theta_{-}^{0}$ are initialized with a predefined uniform distribution. The samples are repeatedly generated by the primary network, which usually is designed with a fixed format $\left(s^{t},a^{t},r^{t},s^{t+1}\right)$ representing a set of current state, selected action, reward, and next state, respectively. A replay buffer with a predefined size is introduced in our model to store the training samples. During the process, these samples are stored into the replay memory with a first-in first-out sequence.

Training: Once the replay memory is filled with the training samples, at each subsequent iteration, a mini-bunch of training samples are uniformly drawn from the replay memory for the model training. During the process, the newly generated tuples are continuously stored into the replay memory to provide up to date samples. Based on the next state $s^{t+1}$ in the tuple, the primary network greedily choose the optimal action $a^{t+1}$ for the next time slot. Thereafter, we can obtain the target *Q*-value with $s^{t+1}$ and $a^{t+1}$ as (26), which can be regraded as the accumulative reward value in one episode. The loss function (27) is utilized to update the primary network’s parameters, by which the network parameter θ are updated along with the negative gradient direction to minimize the loss function as (28). For each $T^{\prime }\left(T^{\prime }<T\right)$ times, the primary network’s parameters are copied to the target network. In fact, the parameters update of the target network is always slower than the pace of the primary network, which can endow a relative stable network training environment. In addition, to reduce the computation complexity caused by the extraordinary huge solution space, the asynchronous updating mechanism can enhance the training stability significantly.

Iteration Stop: For one agent, once the iteration times have reached the specified maximum number of iterations and the value of loss function converges to a small range of values, the training process stop and the action corresponding to the optimal Q-value is chosen as the optimal action. With the asynchronously implement among agents, the optimal computation offloading decision can be obtained sequentially. The main steps for DDQN-based computation offloading management in a CVN is summarized in Algorithm 2.

(26) $$y^{t}=R\left(s^{t},a^{t}\right)+\gamma Q\left(s^{t+1},{\left(a^{t+1}\right)}^{*},\theta_{-}^{t}\right),{\left(a^{t+1}\right)}^{*}=\arg\max_{a^{t+1}}Q\left(s^{t+1},a^{t+1},\theta^{t}\right),$$

(27) $$Loss=\frac{1}{2M^{\prime }}\sum_{1\leq m\leq M^{\prime }}{\left[y^{t}-Q\left(s^{t},a^{t},\theta^{t}\right)\right]}^{2},$$

(28) $$\nabla_{\theta^{t}}Loss=-\frac{1}{M^{\prime }}\sum_{1\leq m\leq M^{\prime }}\left[y^{t}-Q\left(s^{t},a^{t},\theta^{t}\right)\right]\nabla_{\theta^{t}}Q\left(s^{t},a^{t},\theta^{t}\right).$$

**Algorithm 2** DDQN algorithm for Computation Offloading Management in a VCN**Input:** One primary Q-network structure and one target Q-network and one replay memory *M* with size *m***Output:** The optimal computation offloading management solution $\pi^{*}$ 1: Initialize network parameters $\theta^{0}$ and $\theta_{-}^{0}$ of primary network and target network 2: **for all**
$\;t\;\in \;\left(1,\;\cdots ,\;T\right)\;$
**do**
 3:  Generate the current state $s^{t}$ based on the up to date environmental information 4:  Select a action $a^{t}$ based on ε−greedy policy 5:  Obtain reward $r^{t}$ from the environment and transfer to the new state $s^{t+1}$ 6:  Obtain the reward and store the tuple $s^{t},a^{t},r^{t},s^{t+1}$ into memory *M* 7:  **if**
$t\geq C_{memory}$ and the reply buffer is filled up **then** 8:   Draw a mini-batch of tuples $M^{\prime }$ from the reply buffer for model training 9:   Compute the target Q-value by target network with the current state in $M^{\prime }$ 10:   Choose the action greedily with the optimal *Q*-value 11:   Compute the loss function value with (27) and update current Q network with (28) 12:   **if**
$\;t\;=\;nT^{\prime },\;n\;\in \;N^{+}$
**then**
 13:     Update the network parameters of target network with $\theta^{t}\leftarrow \theta_{-}^{t}$ 14:   **end if**
 15:  **end if**
 16: **end for** 17: **return** the final resource allocation result $a^{*}$


## 6. Simulation Results and Analysis

In this section, a host of simulation results are presented to evaluate the proposed algorithms’ effectiveness, which mainly embrace the simulations for LSTM-based resource discovery and the DDQN-based computation offloading management, respectively. To conduct the simulations, we consider a one-way road segment with three lanes. There are different requirements for safety distances and driving speeds for each lane. RSUs are deployed every 500 m on the roadside. An RSU is empowered with a coverage radius of 500 m. The arrival of service vehicles and task vehicles obeys the Poisson process with parameter $\lambda_{s}$ and $\lambda_{t}$, respectively. Each vehicle is equipped with resource availability and resource requirement with the amount of 1 or 2 RUs, respectively. More details will be given in the following, and the other detailed parameters are summarized in Table 1.

### 6.1. LSTM-Enabled Resource Discovery Algorithm

In this section, the effectiveness of LSTM-based resource discovery scheme is verified, and the training and testing stages are, respectively, discussed in the following. At the beginning, we firstly generate a number of samples with the proposed self-similar traffic simulator, and then the whole data set is divided into training data set and testing data set two parts. In our proposed LSTM model, we formulate a four-layer network with two hidden layer. The dimension of the input layer and the output layer are 1×64 and 1×1, respectively. For the two hidden layers, there are 64 cell units stacked in each layer. The training process is implemented for 3000 iterations, and the testing process is performed for 1000 iterations.

**Figure 5:**
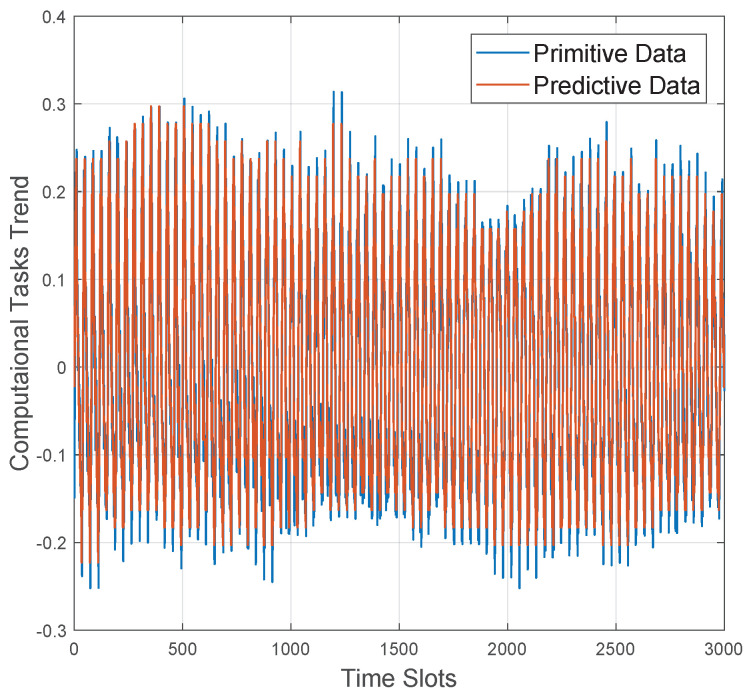
The training stage for LSTM-based resource availability prediction.

Here, an accumulative version of 100 self-similar processes generated by the ON-OFF simulator, is utilized to approximate the variability of one vehicle’s on-board resource utilization status. In this case, Figure 5 represents the training stage’s performance. Specifically, the blue line is the original data generated by the SS simulator, while the red line denotes the predictive results by the proposed LSTM algorithm. It is obvious that the predictive results can perfectly trace the tendency of original data. In Figure 5, the contour of the original data is an approximately irregular wavy line shape, our prediction results can almost replicated its trend very well. Although there are some bursts, the entire data steam’s tendency is not chaotic at all. In fact, the above performance is determined by the fact that the bursts are usually caused by one or a small number of computational processes, which only accounts for a relative small part of the entire data flow. As a result, the bursts caused by a small part of the computational processes cannot dominate the overall computation load trend. As shown in the simulation results, the entire data flow shows potential stability as a whole.

**Figure 6:**
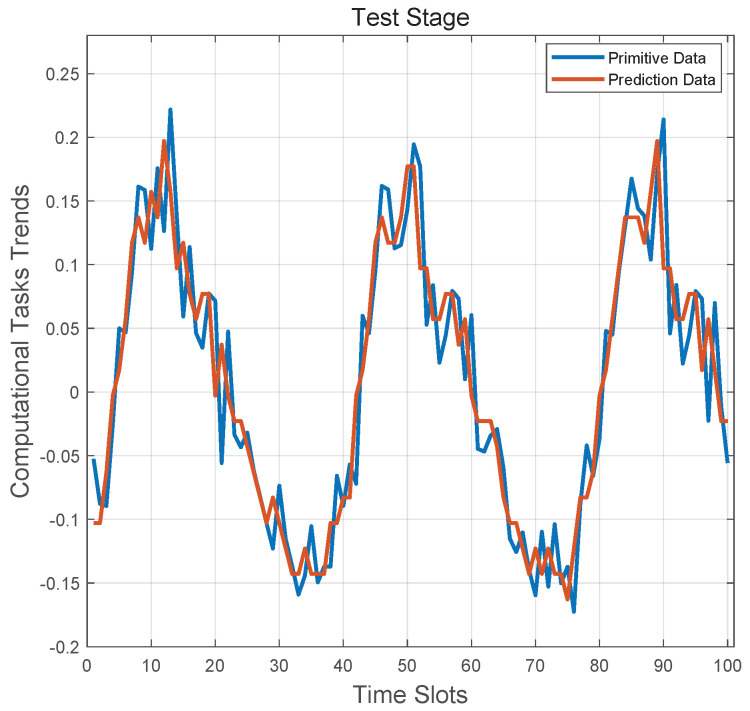
The test stage for LSTM-based resource availability prediction.

Figure 6 evaluates the LSTM-based resource discovery scheme’s performance on testing stage. It is clear that the LSTM algorithm can almost reproduce the entire vehicular tasks flow’s tendency. In order to get a more visual display, we define a criterion y=0 as the baseline of judging the resource utilization status on each service vehicle. Specifically, if one service vehicle’s prediction result is above the baseline, it is considered that the resource utilizing state is idle at next time slot; otherwise, the resource utilizing state is busy. Furthermore, the physical meaning of resource availability prediction curve is depends on the system model. The predictive curve can be discretized with different quantitative steps, and the baseline can be flexibly adjusted to represent more detailed resource utilization status. In this way, the scalability of the proposed LSTM algorithm can be significantly enhanced.

### 6.2. DDQN-Based Computing Offloading Algorithm

**Figure 7:**
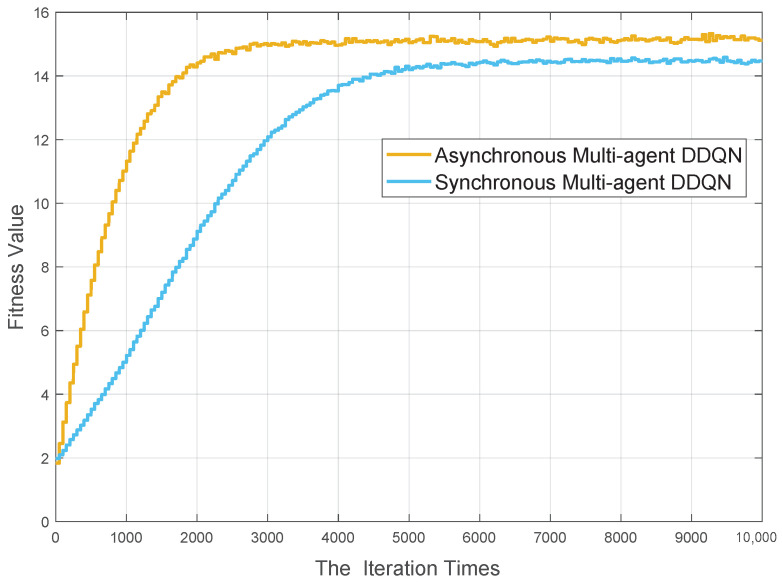
The convergency process of multi-agent DDQN algorithm.

In this section, we verify the effectiveness of the proposed computation offloading management algorithm from different perspectives. During the process, a multi-agent DDQN algorithm with the centralized training and decentralized execution framework in Reference [57] is introduced as a comparative approach, which is named as Synchronous Multi-agent DDQN. In Figure 5, the convergency performance of proposed algorithm is presented with the tendency of loss value. Specifically, the convergence performance can be guaranteed within 4000 iterations. The simulation results have been presented in Figure 7, in which the convergence speed of the proposed algorithm is faster than the synchronous one. The reason is mainly from two aspects, one is the benefits from the designed asynchronous iteration sequence, and the other is the action space consisting of the neighboring service vehicles. Specifically, the asynchronous iteration sequence mechanism endows each agent a relative stable training environment, which can accelerate the convergence speed to the optimal solution. In terms of the reshaped action space, it can provide an action space with a smaller size. A smaller size solution space can definitely cause a faster convergence speed.

**Figure 8:**
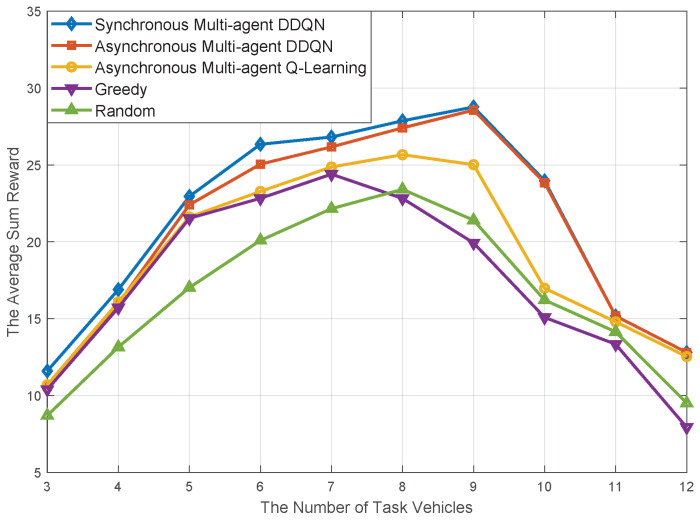
The performance of the overall reward.

In Figure 8, to evaluate the performance of the average overall reward, in addition to the Synchronous Multi-agent DDQN algorithm, we also compare our proposed algorithm with Asynchronous Multi-agent Q-learning, greedy algorithm (Greedy), random algorithm (RANDOM). It is apparent that, except the Synchronous Multi-agent DDQN algorithm, the proposed algorithm’s performance is better than the designed comparative schemes. In fact, owing to the global solution space consisting of all service vehicles, the Synchronous Multi-agent DDQN can obtain a better solution than our proposed algorithm. However, this very limited performance advantage is at the expense of expensive computational complexity, which will be analyzed in detail at the final of this section. From the macro perspective, the overall performance gap between our proposed algorithm algorithm and other comparative algorithms, shows a trend of increasing first and then decreasing. The performance is due to the fact that, with the increase of task vehicles, the resting resource availability provided by service vehicles gradually reduces. During the process, the dimensionality of solution space reduces in a gradual way, and the performance gap between our proposed algorithm and RANDOM get smaller gradually.

**Figure 9:**
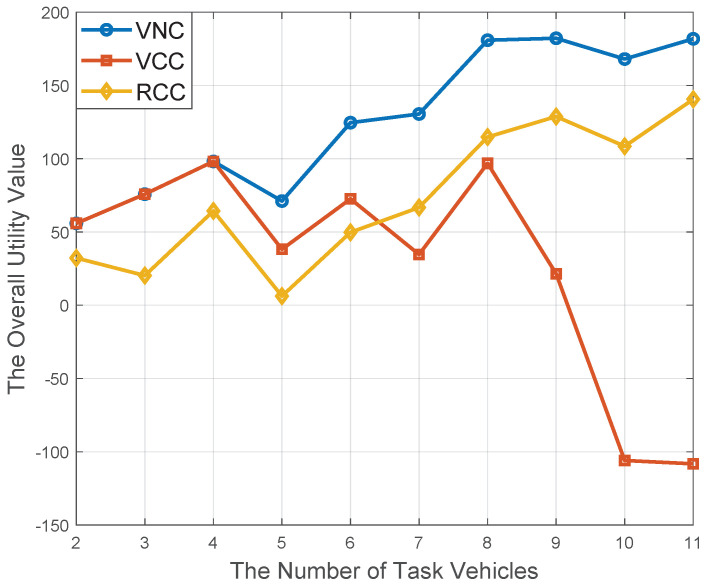
The performance of cognitive vehicular networks (CVN), vehicular cloud computing (VCC), and remote cloud computing (RCC) schemes.

In Figure 9, the performance of the proposed computation offloading scheme (CVN) is simulated, which joins VCC and RCC together. The computation offloading schemes enabled by VCC and RCC are utilized as the comparative schemes. It is apparent that CVN scheme can achieve better performance than both VCC and RCC schemes. At the beginning, there are less task vehicles in the model and the service vehicles’ on-board computation resource is sufficient to accommodate the task vehicles’ resource requirements. In this way, CVN scheme and VCC scheme can achieve the same performance. With the increase of task vehicles, the resource availability of service vehicles cannot satisfy their resource requirements any longer. As a result, CVN scheme demonstrate a better performance than the VCC scheme. Due to the expensive price of computation resource provided by RSUs, the performance of RCC scheme is always inferior to CVN scheme.

**Figure 10:**
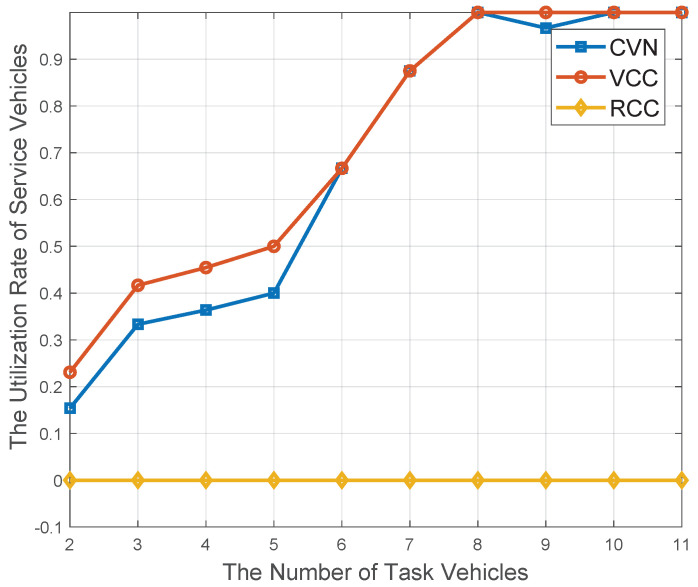
The resource utilization rate of service vehicles between CVN, VCC, and RCC schemes.

With the identical simulation environment with Figure 9, the resource utilization of service vehicles is shown in Figure 10. In particular, the resource utilization [36] can be defined as the ratio of the allocated resource to the overall on-board resource availability. From Figure 10, it is obvious that VCC scheme can achieve better performance than CVN and RCC algorithm. It is obvious that there is no other available computational resource except the underutilized on-board resource in the VCC scheme. In this way, the VCC scheme’s utilization rate is the highest one than the other schemes. In contrast, for the RCC scheme, all the computation tasks are offloaded to resource-rich RSUs, and service vehicles’ underutilized on-board resource are not utilized at all; thereby, its utilization rate is the lowest one.

**Figure 11:**
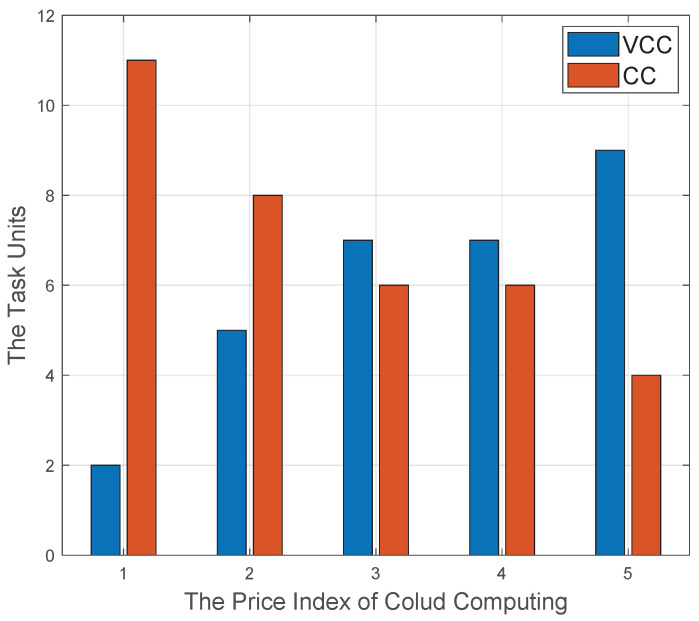
The resource utilization distribution between VCC and RCC with the increasing price of computation resource on RCC.

Under a total 13 computation tasks, Figure 11 presents the impact of the pricing strategy on the computation offloading management scheme. Specifically, the computation tasks allocated to service vehicles’ on-board resource or cloud computing center are defined as VCC and CC, respectively. The price index of cloud computing corresponds to a set of gradually increasing unit price. It is apparent that, with the increase of unit price of RCC, the resource utilization from RCC decreases gradually. It is natural that task vehicles tend to choose cheaper on-board computation resource. In other words, if the pricing strategy of RCC is reasonable, the RCC-based computation offloading will be welcomed by task vehicles.

**Figure 12:**
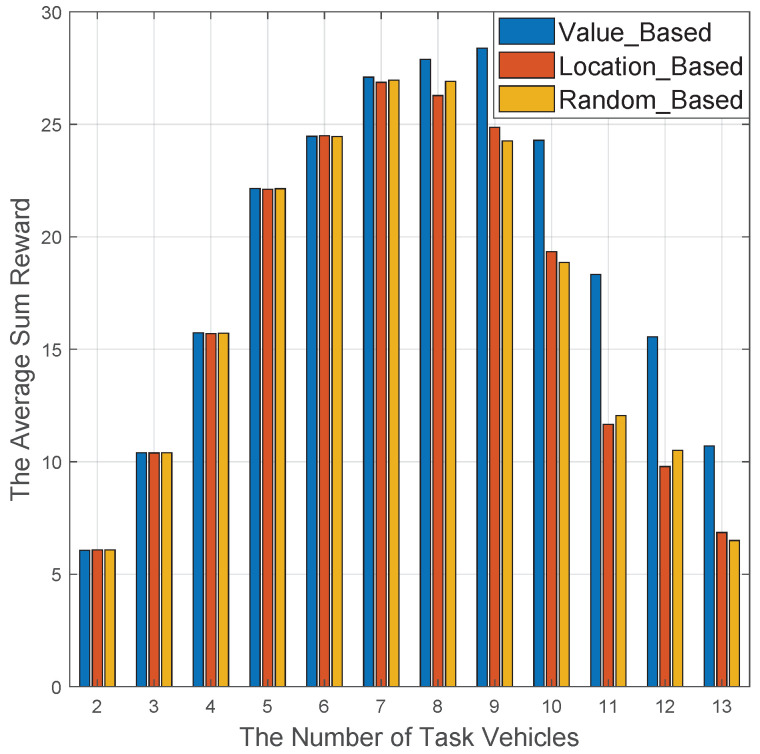
Performance comparisons among different iteration orders.

Under a vehicular scenario with 15 vehicles, the performance comparisons among Value-based, Location-based, and Random-based asynchronous iteration sequence are shown in Figure 12. It is obvious the different stages demonstrate heterogeneous tendencies. In the first half stage, $K_{t}\leq 7$, the three schemes’ performance are almost identical, whereas the Value-based scheme is better than the other schemes. The reason is due to the fact that the case $K_{t}\geq 8$ means that there is less resource availability than resource requirement in the VCC network, and the Value-based scheme can give priority to the task vehicles with high-value, which certainly enhances the overall performance than other schemes. In addition, the Location-based scheme and random-based scheme can achieve a similar performance since they neglect the value information in the resource scheduling process in the VCC network.

**Figure 13:**
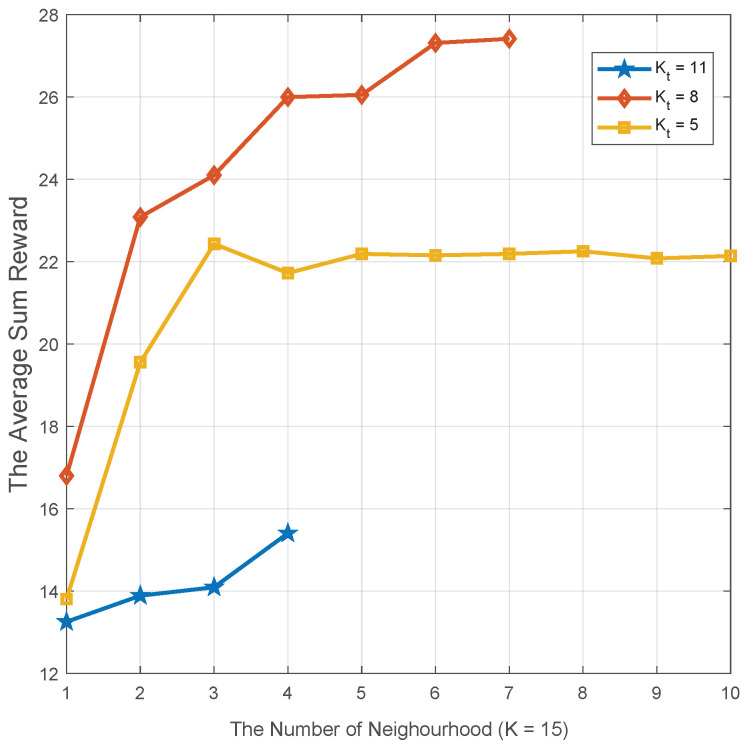
Performance comparisons with different neighbors.

In Figure 13, the impact of the number of neighbor agents on system performance is evaluated. In a CVN with 15 vehicles in total, three sizes of neighboring services vehicles: large (11), intermediate (8), and small (5), are simulated, respectively. Moreover, in addition to service vehicles, all other vehicles are task vehicles. Based on the certain service vehicles, the size of neighbor agents gradually changes from 1 to 10. It is apparent that the three cases’ overall tendency are gradually rising. In fact, the larger neighborhood space can achieve better environmental information and more available resource options. From a macro perspective, the intermediate scheme can achieve the best performance compared to the other schemes. Specifically, the intermediate size can achieve a relative balance between resource supply and demand. However, for the small size, the available resource is much more than task vehicles, and only a small part of resource availability can be utilized. In terms of the large size, the task vehicles’ resource requirements cannot be satisfied by the limited on-board resource availability provided by service vehicles. Generally speaking, although a bigger neighbor agents’ size can obtain a better performance, a bigger size heralds a greater computation complexity. Above all, under the limited computation capability, a reasonable neighbor agents’ size is very important to obtain a better performance.

Last but not least, considering the importance of the computation overhead, we analyze the computational complexity of the proposed computation offloading algorithm. In our model, since the primary network and the target network have identical structures, we only consider the primary network to reflect out model’s computational complexity. In fact, the computational complexity of the primary network mainly comes from the execution stage and training stage. Due to the fact that the backpropagation-based training stage is the reverse process of the execution stage, we only calculate the execution stage’s computational complexity. Specifically, the amount of neurons of the *m*th layer of the primary network is defined as $U_{m}$, and the computational complexity of *m*th layer is $O\left(U_{m-1}U_{m}+U_{m}U_{m+1}\right)$. As a result, for the primary network with *M* layers, the execution stage’s computational complexity is $O_{execution}=O\left(\sum_{2\leq m\leq M-1}\left(U_{m-1}U_{m}+U_{m}U_{m+1}\right)\right)$. Moreover, for each agent, its overall computational complexity is $3\cdot O_{execution}$, which mainly considers the execution stage on the primary network and the target network, as well as the training stage on the primary network. As a result, the overall computational complexity of the propose multi-agent DDQN algorithm is $3\cdot N\cdot O_{execution}$. With the same derivation approach, the computational complexity of the synchronous multi-agent RL algorithm is $3\cdot N^{M}\cdot O_{execution}$; it is obvious that our proposed algorithm with asynchronous iteration order has a much less computational complexity. Furthermore, considering that the synchronous training scheme needs to spend more iterations in the training process, our proposed algorithm’s superiority is more prominent from the perspective of computational complexity.

## 7. Discussion

In this paper, to accommodate the resource-intensive vehicular applications, a computation offloading scheme integrating VCC and RCC was proposed. Specifically, VCC represents on-board computation resource, and RCC indicates infrastructure’s computation. In our model, the VCC is equipped with a dynamic and uncertain resource availability, while the RCC’s resource availability are stationary and sufficient. To overcome the challenges brought by the dynamic and uncertain resource availability of VCC, the concept of CVN is proposed. To solve the computation offloading in a CVN, a perception-exploitation mechanism is designed, which mainly comprise resource discovery and computation offloading two parts. To the best of the author’s knowledge, it is the first work to investigate the computation offloading under the vehicular scenario with a dynamic and uncertain resource availability. In particular, although there have been some on-board computation resource-based computation offloading related works, the dynamics and uncertainty of on-board computation resource are always neglected or it is assumed that as the resource utilizing status can be obtained effortlessly. Due to the privacy concern and selfish attitude, the conventional computation offloading scheme with a clearly known resource utilization status are unrealistic at all. To overcome the resource uncertainty, we propose an LSTM network-based resource discovery mechanism to predict the on-board resource availability. Based on the resource discovery results, a multi-agent DRL algorithm is proposed to obtain the computation offloading strategy, in which multiple agents iteratively update to guarantee the training stability.

From both theoretically and practical perspective, the proposed computation offloading scheme all reflect important significance. Due to the dynamics and uncertainty of resource availability in the VCC, the perception-discovery mechanism is innovatively proposed to conduct the computation offloading in a CVN. In the resource discovery stage, one LSTM network is designed to extract the temporal correlation from the collected historical data and predict the resource utilization status of VCC. The idea is consistent with the prediction algorithms in transportation field, such as the prediction of vehicle speed, vehicle path and road traffic flow, etc. From the theoretical perspective, the LSTM network is successfully extended to predict the on-board resource utilization status prediction, and its effectiveness in extracting the temporal correlation from the historical data has been effectively verified with the simulation results. Moreover, the proposed computation offloading management scheme has important practical implications in the domain of vehicular network, especially in a vehicular environment with high communication costs or poor channel quality. In addition, another important aspect of our proposed solution is its efficiency on computation offloading management. Specifically, with the proposed resource discovery scheme, we can conduct the computation offloading management in advance. Furthermore, the proposed computation offloading management algorithm is an asynchronous iterative algorithm, and the neighbor vehicles-based solution space is reduce to the computation complexity, as well. More scheduling time can be saved to achieve an efficient computation offloading management. As a result, from the practical perspective, the proposed computation offloading management scheme can be applied to delay-sensitive vehicular applications. Furthermore, in our work, some important conclusions have been drawn. The pricing mechanism has also been proven an important factor to influence the final computation offloading strategy. Furthermore, the setting of the iteration sequence and the size of neighbor agents-based action space can have a greater impact on the final system performance. Nevertheless, there are still many unsolved issues in our investigation and a handful of open problems worth future research efforts. Owing to the shortage of the realistic training data, the proposed scheme’s effectiveness should be further verified. Moreover, the proposed computation offloading scheme has not considered the QoS of vehicular applications. These issues will be studied in our future research.

Frankly speaking, in this work, due to the limitations of our current research, the proposed perception-exploitation mechanism only involves the prediction of the utilization status of on-board computing resources. However, the proposed scheme can be easily extended to other more complex issues, such as the size of the vehicle tasks and the safety status of vehicles, etc. To solve the computation offloading with some factors in dynamics and uncertainty, the core issues are to find a suitable statistical analysis model and design a state prediction model for the statistical analysis model. The size of computation tasks usually refers to two aspects: the data size and computation complexity. In most studies, the computation complexity is proportional to the data size with a predefined ratio. In this way, The size of the vehicular task can uniquely determine the computation complexity. Under the assumption that the computational complexity can be uniformly quantified into multiple levels, the vehicular tasks with different size can be regarded as an accumulative version of multiple sub-task with a smallest level of computation complexity. In this way, the proposed resource discovery scheme in this paper can be scaled to the computation offloading with heterogeneous size of vehicular applications. Moreover, in the open vehicular environment, the security of vehicles is often threatened. Specifically, some vehicles are harmfully used by hackers and other malicious criminals. For example, some malicious vehicles pretend to be service vehicles to illegally steal important privacy information from other vehicles, while some vehicles disguise themselves as task vehicles to spread false information or deliver computing tasks that contain viruses. In this way, the safety status is an important factor for reliable computation offloading in vehicular network. To conduct the safety computation offloading, an effective safety status prediction mechanism should be designed. In fact, the safety status can be reshaped as a sequence of 0–1 value. Based on the collected historical trust status information, the safety status can be predicted by a customized predictive model. Furthermore, blochchain technology can be integrated into the predictive model to enhance the robustness performance. These new ideas will be studied in our future research.

## 8. Conclusions

In this paper, we investigate the computation offloading in a cognitive vehicular network (CVN), which jointly considers the on-board computation resource on vehicle cloud computing (VCC) and computation resource equipped in the remote cloud computing (RCC) center. The overall scheme mainly comprises resource discovery and computation offloading management two stages. As for resource discovery, a Long Short-Term Memory (LSTM)-based resource discovery algorithm is proposed to solve the dynamics and uncertainty of VCC’s resource availability. Moreover, to generate the training and testing data sets, a self-similar simulator is introduced in our model. Based on the obtained resource discovery results, a multi-agent double deep Q-learning algorithm is adopted to implement the computation offloading management, in which an asynchronous iteration mechanism is designed. To verify the proposed algorithm’s effectiveness, a host of experimental simulations results are presented in our work.

## Figures and Tables

**Table 1:** System simulation parameters.

Parameter	Value
Cellular transmission power	0.2 W
Baseline power for V2mV link	0.1 W
Noise power	−174 dBm/Hz
Pathloss index	2
Number of Lanes	3
Velocity of Each Lane	[120 km/h, 90 km/h, 60 km/h]
Safety Distance of Each lane	[120 m, 90 m, 60 m]
Lane width	4 m
Bandwidth of Each Vehicle	20 MHz
Power allocation index	0.8, 0.2
Learning rate α	0.8
